# Cystic Echinococcosis of the Ilium Treated with Curettage and Microwave Thermoablation Followed by Bone Cement Installation: A Novel Treatment Technique for a Rare Disease

**DOI:** 10.1155/2021/5533183

**Published:** 2021-06-24

**Authors:** Kyriakos Papavasiliou, Sousana Panagiotidou, Panagiotis Kakoulidis, Antonia Bintoudi, Kostoula Arvaniti, Eleftherios Tsiridis

**Affiliations:** ^1^Academic Orthopaedic Department, Papageorgiou General Hospital, Aristotle University Medical School, Thessaloniki Ring Road, 56403 Nea Efkarpia, Greece; ^2^Department of Radiology, Papageorgiou General Hospital, Thessaloniki Ring Road, 56403 Nea Efkarpia, Greece; ^3^Intensive Care Unit, Infection Control and AMS Unit, Papageorgiou General Hospital, Thessaloniki Ring Road, 56403 Nea Efkarpia, Greece

## Abstract

Bone cystic echinococcosis (CE) is a rare condition requiring a high level of suspicion during primary diagnosis. Wide excision of the lesion is the gold standard of treatment, posing however extreme challenges in certain parts of the skeleton, since it may well be accompanied by increased morbidity. We report the case of a 35-year-old Caucasian female with iliac bone CE, referred to our department (a regional referral center for the treatment of patients with musculoskeletal tumors). The patient reported gradually increasing dull pain at the right iliac fossa and antalgic gait, with an onset of approximately 5 years before her referral. Bone CE diagnosis was established based on physical examination, imaging studies, and two subsequent CT-guided core needle biopsies, performed within a period of 3 months, of which the second was diagnostic. Following a musculoskeletal tumor multidisciplinary meeting, it was decided that the optional treatment was the surgical removal of the cyst. Aiming to minimize the morbidity accompanying a wide resection of the lesion, we performed extended curettage of the lesion through a typical iliac spine approach, followed by microwave ablation of the walls of the remaining bone cavity. The remaining iliac defect was treated with the installation of polymethyl methacrylate bone cement. The patient reported immediate remission of symptoms postoperatively and was able to return to everyday activities two weeks postoperatively. She began oral treatment with albendazole on the 7th postoperative day. She remained symptom-free for a period of 25 months, until she developed a seroma at the gluteal area, which was treated with simple drainage. On her latest follow-up six months later, she remained symptom-free and was able to perform all her previous activities. Microwave ablation may serve as a useful adjuvant modality when treating patients with bone CE, in order to prevent relapse of the disease.

## 1. Introduction

Cystic echinococcosis (CE) is a parasitic infection caused in humans by *Echinococcus granulosus* sensu lato [1]. It mainly affects the liver and the lungs, while occurrence at skeletal parts is rather rare [2]. The spine, pelvis, and femur are the most common locations of CE or hydatid cysts at the skeleton, with a reported incidence ranging from 0.5% to 4% of patients with CE [2]. Treatment of bone CE is challenging, since the radical excision of the parasite is a rather difficult task, even after the wide resection of a lesion [3]. As a result, the rate of recurrence following surgical treatment is rather high, reaching 48% of cases [4].

We present the case of an unusual location of bone CE at the ilium, treated with intralesional curettage and microwave ablation, followed by the installation of polymethyl methacrylate (PMMA) bone cement into the remaining cavity to prevent postoperative fracture and potential relapse of the lesion. To the best of our knowledge, this is the first case in the English literature, presenting this novel adjuvant treatment modality as a means of treatment for bone CE.

This case has been reported according to the SCARE 2018 criteria [5].

## 2. Case Presentation

A 35-year-old Caucasian female was referred to our department (a regional referral center for the treatment of patients with musculoskeletal tumors), complaining about ongoing pain at her right ilium and intermittent limping for the past 5 years. Symptoms were not initiated by trauma. The patient had been working in a food processing factory for approximately 13 years and was the owner of a dog for a couple of years. Her medical history revealed an echinococcal cyst of the liver diagnosed 3 years before her referral and during investigation for anemia. She was on a regular follow-up schedule by general surgeons.

**Figure 1:**
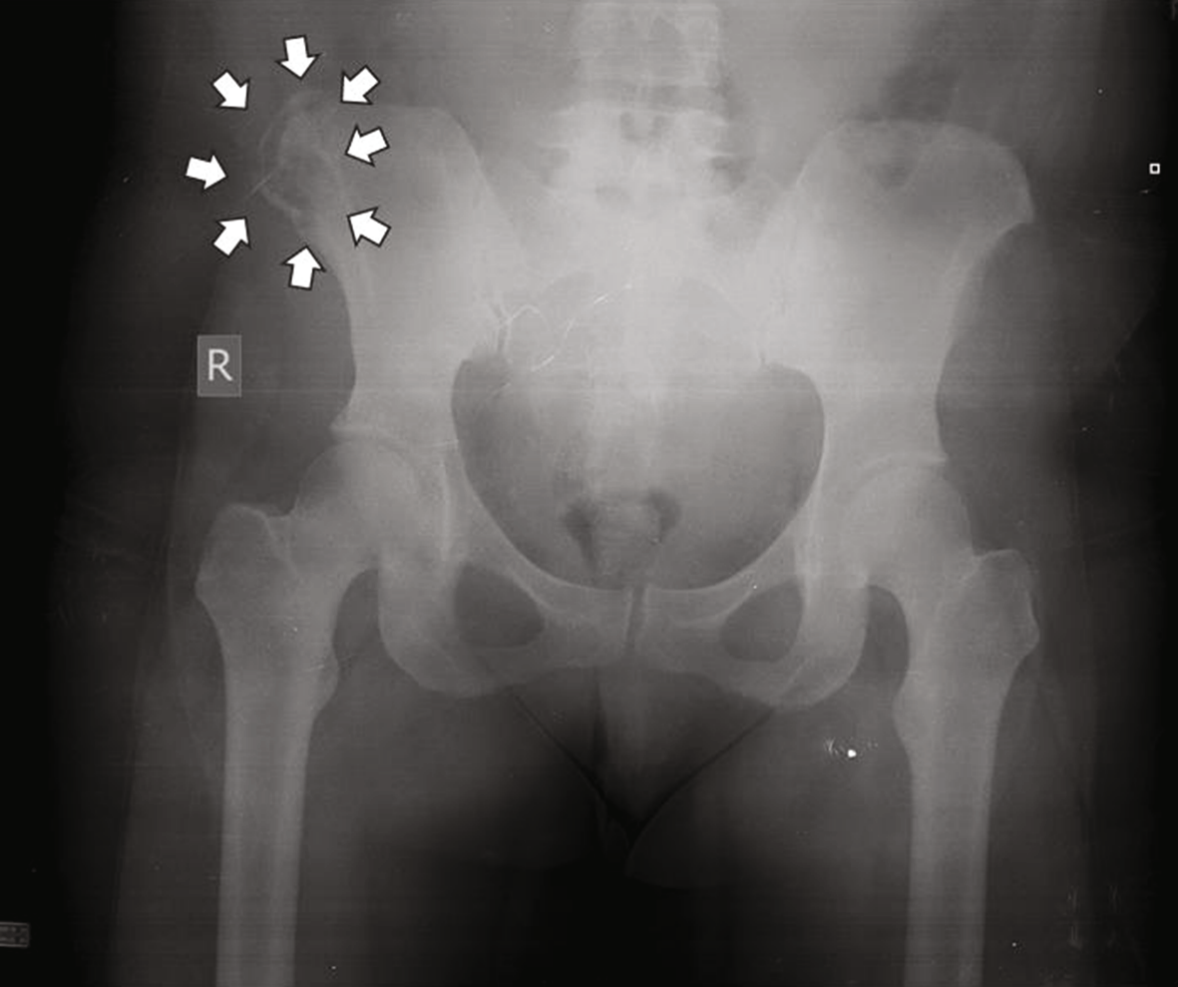
The initial standard anteroposterior radiograph of the patient's pelvis, depicting a lytic lesion (white arrows) at the iliac crest of the right ilium.

**Figure 2:**
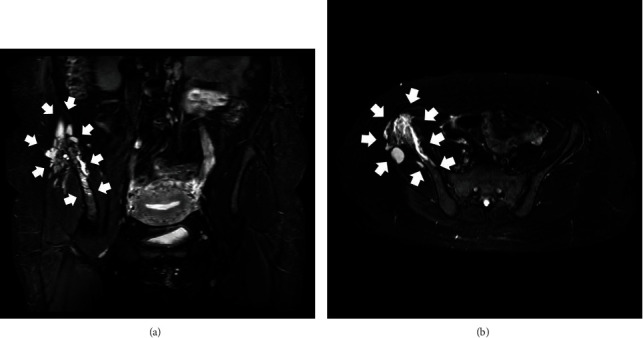
The initial MRI scan of the patient's pelvis. Short TI Inversion Recovery-weighted image sequence (STIR) coronal (a) and axial (b) views. Both pictures demonstrate a lesion with extended bone edema and nonhomogenous appearance, originating from the area of interest along the right iliac crest, combined with extension to the adjacent soft tissue structures (white arrows).

**Figure 3:**
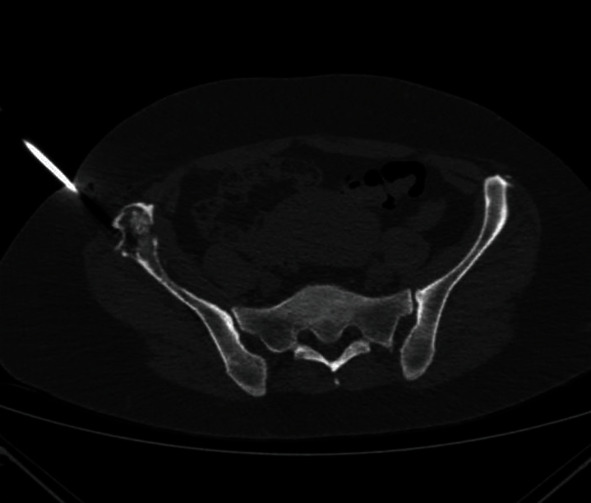
CT scan of the patient's pelvis (axial view) depicting the CT-guided core needle biopsy of the lesion at the right iliac crest.

**Figure 4:**
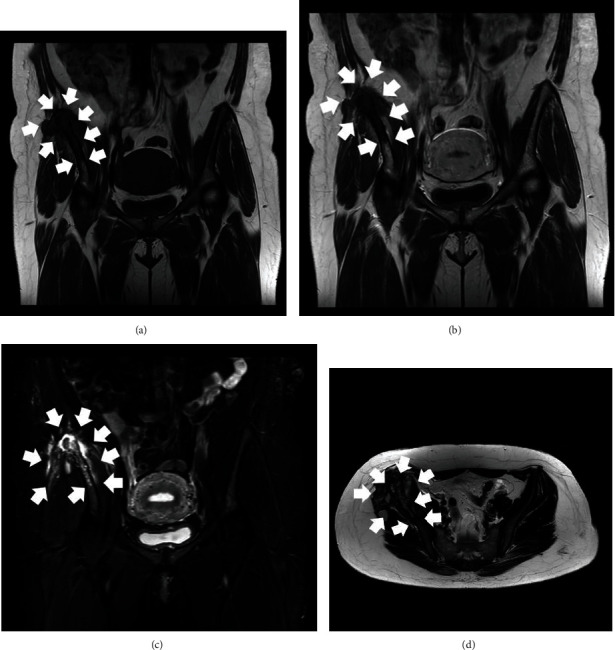
MRI scan of the patient's pelvis three months after her initial evaluation. T1-Weighted Image (T1W) coronal view (a), T1W contrast-enhanced coronal view (b), STIR coronal view (c), and T2-Weighted Image (T2W) axial view (d). The lesion appears to have progressed compared with the initial MRI scan evaluation and continues to be characterized by atypical imaging features, edema, and mixed osteolytic and osteoblastic areas (white arrows).

Upon clinical examination, the patient had antalgic gait and reported local tenderness elicited on palpation at the right ilium. The passive hip range of motion was normal, and no leg length discrepancy was detected. Further investigation with a plain radiograph of the pelvis (Figure 1) and Magnetic Resonance Imaging (MRI) (Figure 2) performed upon her first visit revealed an atypical lytic lesion at the right ilium. Laboratory tests showed a mild increase of the Erythrocyte Sedimentation Rate (ESR) (26 mm/hr) and of the C-Reactive Protein (CRP) (1.07 ng/L, normal range < 0.5 ng/L). The patient subsequently underwent a Computed Tomography- (CT-) guided core needle biopsy (Figure 3) with a pathology report negative for malignancy yet nondiagnostic. The patient was given instructions for crutch-assisted partial weight bearing and reevaluation. Three months later, a second MRI scan was performed (Figure 4) and showed further destruction of the right ilium crest, with the presence of extended osteolytic areas. A second CT-guided biopsy followed, and the pathology report was positive for bone CE of the ilium.

**Figure 5:**
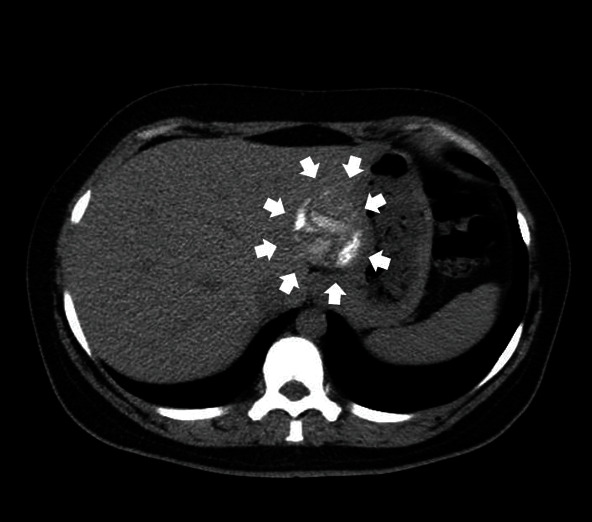
Contrast-enhanced CT scan of the abdomen (axial view) three months after the initial evaluation of the patient. Notice the existence of a single hydatid cyst with distinct calcified walls, arising from segment II of the left lobe of the liver (white arrows).

**Figure 6:**
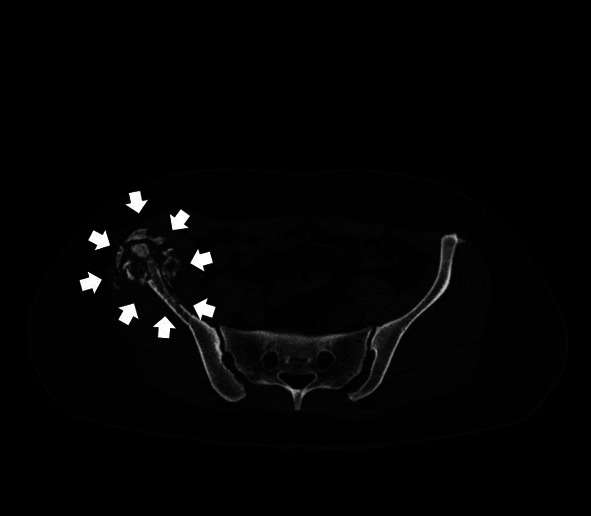
CT scan of the pelvis (axial view) of the patient three months after her initial evaluation. Notice the large cystic lesion at the iliac crest, with signs of the destruction of the ilium and areas of calcification (white arrows).

The patient next underwent full staging. A contrast-enhanced CT scan of the abdomen revealed a 6.4 × 4.5 × 3.3 cm calcified cystic lesion (Figure 5). A CT scan of the pelvis depicted a large cystic lesion at the iliac spine with osseous destruction of the right iliac bone anatomy (Figure 6). Following a musculoskeletal tumor multidisciplinary meeting with the ad hoc presence of an infectiologist and a general surgeon, it was decided that the optional treatment ought to be the surgical removal of the cyst and this should precede any other treatment, mainly due to the patient's ambulatory problems. Due to the location of the bone CE at the iliac crest and its size, and in order to prevent the increased morbidity that would accompany a wide excision, it was decided to implement a complex three-stage novel treatment technique, described as follows.

### 2.1. Surgical Technique

**Figure 7:**
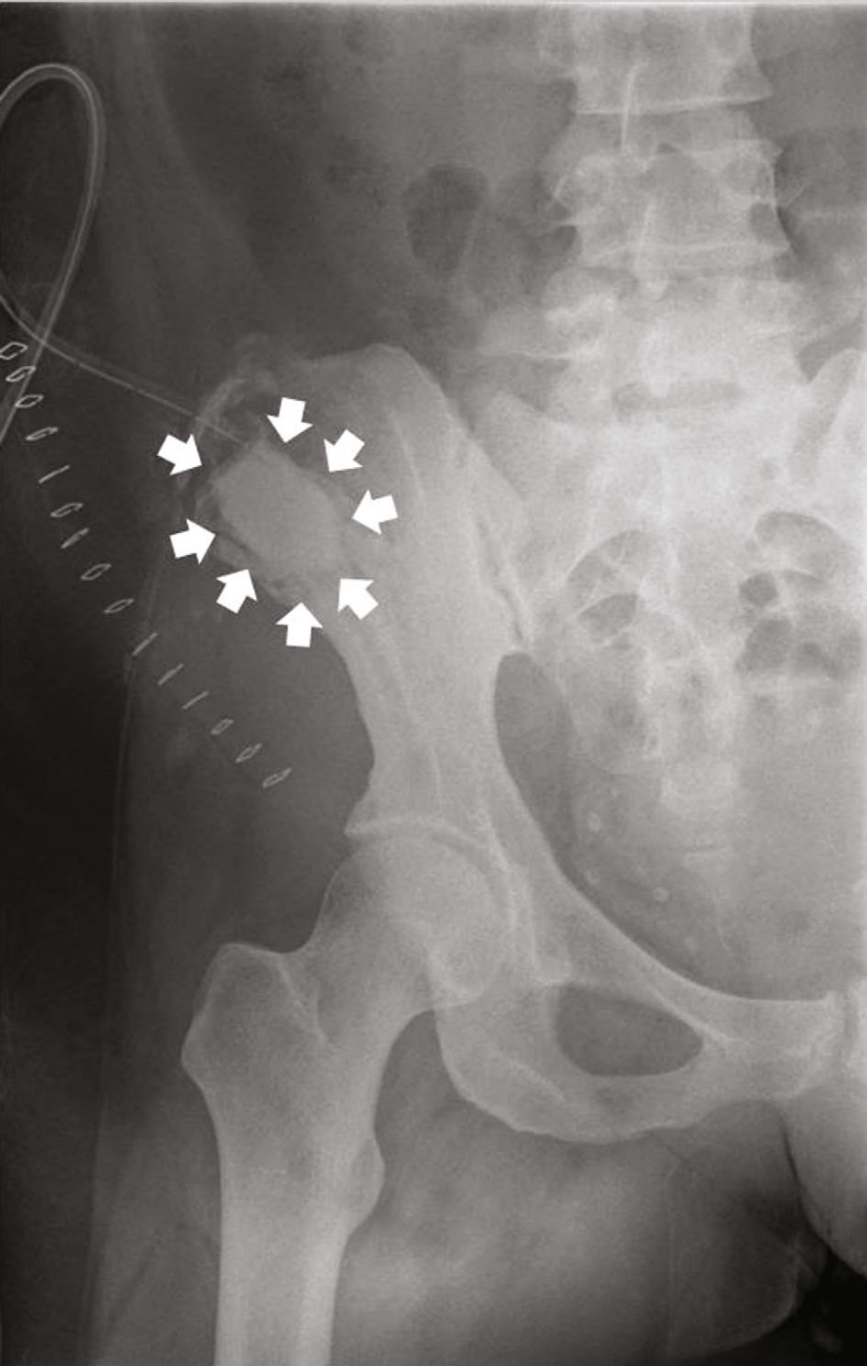
Immediate postoperative anteroposterior radiograph of the patient's hemipelvis, following the curettage of the cyst and the installation of PMMA at the remaining bone cavity (white arrows).

Under epidural anesthesia and following standard antibiotic prophylaxis administration (cefoxitin 2 gr IV q8hr for 24 hrs), with the patient in the supine position, we performed a standard incision along the iliac crest. The subcutaneous fat and the fascia were next incised, giving direct access to the right ilium and to the cyst. We then performed careful and extended curettage of the bone CE (stage 1), taking careful preventive measures to avoid the contamination of the surrounding tissues with the cyst's liquid content (the operative area was meticulously covered with compresses immersed in 20% hypertonic saline). No viability of the parasite could be established in the products of the curettage, which nonetheless reconfirmed the diagnosis of bone CE. This was followed by the microwave thermoablation of the cyst's walls (stage 2), which was facilitated with the introduction to the bone cavity of an Amica™ (HS Hospital Service S.p.A., Rome, Italy) microwave thermoablation probe (16 gauge × 150 mm, combined with a mini choke and an internal water cooling system). The calculated total microwave output was 60 W at 2450 MHz, and the duration of the ablation was 6 min. The remaining bone cavity was filled with the installation of polymethyl methacrylate (PMMA) bone cement (stage 3) in order to prevent the potential development of a postoperative fracture and to prevent the potential relapse of the bone CE (Figure 7). The wound was closed in layers, and a simple vacuum drain was used.

The patient's postoperative course went uneventfully. The patient began crutch-assisted nonweight bearing ambulation on the first postoperative day. The drain was removed on the second postoperative day, and she was subsequently discharged. On the seventh postoperative day, the patient commenced oral treatment with albendazole 400 mg q12hr. Crutch-assisted gradually increasing weight bearing ambulation begun at the second postoperative week. The patient was able to walk with no assistance at two months postoperatively, when she was also able to return to her usual everyday activities.

**Figure 8:**
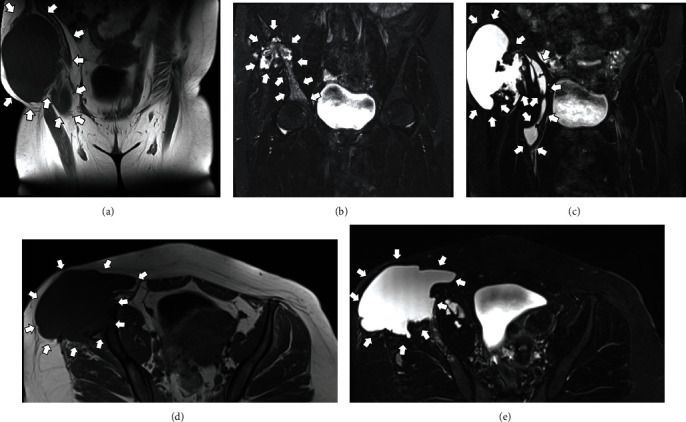
MRI scan of the patient's pelvis 25 months postoperatively. T1W coronal view (a), T2W coronal views (b, c), T1W axial view (d), and T2W axial view (e). Notice the extended Morel-Lavallée-type cystic lesion at the right gluteal area, with no sign of direct contact with the area where the cystic echinococcosis used to be at the iliac crest.

**Figure 9:**
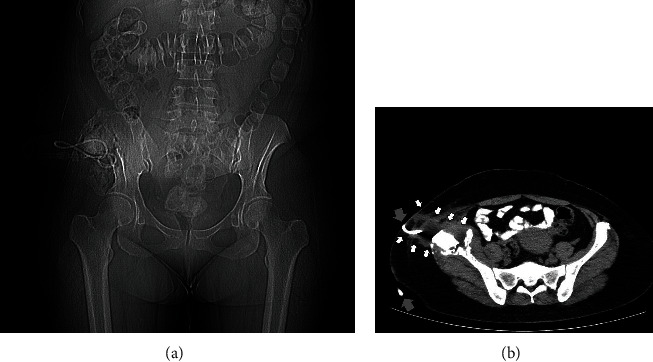
CT scanogram of the patient's abdomen and pelvis (a) and CT scan (axial view) of the patient's pelvis (b) depicting the CT-guided insertion of the 14-gauge drainage catheter (gray arrows) into the Morel-Lavallée-type cystic lesion at the right gluteal area.

**Figure 10:**
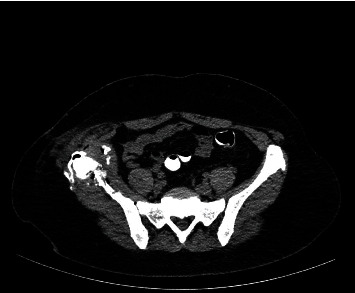
CT scan of the patient's pelvis (axial view) following the removal of the catheter that was used in order to drain the Morel-Lavallée-type cystic lesion at the right gluteal area, confirming the complete drainage of the cystic lesion.

She remained symptom-free for almost 25 months following her operation, until she returned complaining about pain at the operated area. An MRI scan (Figure 8) showed a Morel-Lavallée-type lesion located at the ipsilateral gluteal area, although she did not report any injury whatsoever. The patient underwent uneventful CT-guided drainage of the seroma. Under local anesthesia and CT guidance, a 14-gauge catheter was inserted at the cystic lesion (Figure 9). Culture swabs obtained from the drained fluid came back negative for bone CE recurrence and/or microbial infection. The catheter remained in place for a period of 10 days, in order to facilitate the complete drainage of the cyst and to avoid its recurrence. While the catheter was in place, the patient was under antibiotic prophylaxis (cefuroxime 500 mg PO q12hr) and fully ambulant. The catheter was removed uneventfully, and a subsequent CT scan confirmed the complete drainage of the cyst (Figure 10).

On her latest follow-up visit, at 31 months postoperatively (6 months following the seroma's drainage), the patient remains symptom-free, is still under oral antiparasitic therapy, has no signs of recurrence of the bone CE and of the seroma, and is scheduled to undergo hepatic surgery.

## 3. Discussion

Bone CE lesions are caused by the parasitic tapeworm *Echinococcus granulosus* [1, 2] and mainly occur in the trabecular bone, eventually extending to subcortical areas as the disease progresses [6]. They usually present with atypical imaging features and laboratory tests, hence posing a diagnostic problem and thus delaying treatment in early stages [6]. Differential diagnosis may include tumors and tumor-like lesions of the bone, tuberculosis, and fungal infections [7]. Echinococcal bone lesions may be unifocal or appear in multiple locations [2]. Most patients remain asymptomatic until the advanced stages of the disease, when symptoms like local pain and tenderness first appear [1]. Laboratory tests may reveal an elevated ESR, CRP concentration, and eosinophil count [8]. A positive antibody titer may also be helpful in the diagnosis [8].

The radiographic findings of bone CE are nonspecific [9]. Plain radiographs and CT scans may reveal a single or multiple osteolytic area(s), cortical thinning, calcification, and pathological fractures [9]. MRI scans are useful in order to accurately depict the extent of the disease and the potential extension to the adjacent soft tissues [9]. Bone CE can mimic tumors, simple cysts, and infections [8]. Further investigation with CT scans of the brain, lungs, and abdomen is essential while investigating a suspicious bony lesion of this type [10]. The bone biopsy and specimen culture are of utmost importance in the diagnosis of bone hydatid cysts [8, 11].

The gold standard when treating bone CE is the combination of surgical excision and antiparasitic therapy [3, 6]. There is a wide range of operative options, ranging from simple drainage or debridement to complete cyst excision, extensive bone removal, installation of bone grafts and PMMA, osteosynthesis, and arthroplasty [3]. The use of radiofrequency thermal ablation (RFA) as a treatment for hydatid cysts of the liver has been previously reported [12, 13]. The high temperature released in the cyst results in protein denaturation and further destruction of the germinal layer [14].

There are only a few cases of bone CE of the ilium reported in the literature, and they were treated with a variety of operative and nonoperative methods. Gdoura et al. reported a case located at the pelvis, which was treated with hemipelvectomy, followed by femoropubic and sacral arthrodesis [15]. In another case, the authors showed good results with the combination of antiparasitic therapy, wide excision, and the use of PMMA [16]. Emami et al. reported a case of an intraoperatively diagnosed bone CE which was treated with partial iliac wing excision, instead of the scheduled sequestrectomy [17]. Neelapala et al. presented a case treated with total hip arthroplasty and subtotal removal of the bone CE, which later necessitated a revision operation due to component loosening [18]. Akhan et al. treated a patient with a large bone CE of the ilium by implementing the nonoperative percutaneous modified catheterization technique (MoCaT). This was the first time that this technique was used in bone CE, and it was proven successful not only in dealing with the patient's condition but also in obliterating the need for an operation, which would probably have been accompanied by increased morbidity, due to the extension and the location of the lesion [19].

Bone CE presents with increased recurrence rates, especially when radical excision cannot be achieved [20]. In the hereby reported case, we decided to use microwave thermoablation and installation of PMMA, as adjuvant therapies to the extended curettage we implemented in order to avoid the morbidity associated with the wide excision of bone CE, which is usually used in these cases. Microwave thermoablation increases tissues' temperature locally and in an absolutely controllable manner, leading to the thermocauterization of a lesion. The use of PMMA as a primary or adjuvant treatment modality for benign [21] and malignant bone tumors [22] is also a well-established method of treatment. Furthermore, following the curettage of bone CE, the subsequent installation of PMMA in the remaining cavity (which is also associated with locally increased temperature) was also shown to be effective in preventing disease recurrence, due to the necrotizing impact exerted on the parasitic infection. Last but not least, PMMA is also used as a means of stabilization in order to prevent a pathological fracture [3].

The combination of microwave thermoablation with the installation of PMMA seemed to have served well in order to avoid the recurrence of the bone CE. The development of a large seroma at the gluteal area 25 months postoperatively may be possibly attributed to the extended surgical approach implemented and to the preventive measures taken intraoperatively in order to prevent the contamination of the surrounding tissues, although this cause-and-effect model is neither certain nor verifiable. However, it cannot be related to the bone CE itself, since no recurrence could be established, and furthermore, it occurred after two years from its operative treatment.

To the best of our knowledge, this is the first case in the English literature, presenting this novel adjuvant treatment modality as a means of treatment for bone CE. Although rare, bone CE may necessitate operative interventions accompanied by increased morbidity. The implementation of microwave thermoablation, along with other less invasive treatment modalities, may serve as a useful adjuvant technique, in order to increase the possibility of no recurrence, when less radical operative options are used.

## Data Availability

All related data are available on request.
